# Effect of cell density on decrease in hydraulic conductivity by microbial calcite precipitation

**DOI:** 10.1186/s13568-022-01448-0

**Published:** 2022-08-08

**Authors:** Kağan Eryürük

**Affiliations:** Graduate School of Science, Department of Civil Engineering, Necmettin Erbakan University, Konya, Türkiye

**Keywords:** Column, Microbially induced calcium carbonate precipitation, Number of cells, Optical density, *Sporosarcina pasteurii*, Microbial clogging, Glass beads, CaCO_3_, Hydraulic conductivity

## Abstract

The effect of number of cells deposited on decrease in hydraulic conductivity of porous media using CaCO_3_ precipitation induced by *Sporosarcina pasteurii* (ATCC 11,859) was examined in columns packed with glass beads in the range of 0.25 mm and 3 mm in diameter. After resting *Sporosarcina pasteurii* cells were introduced into the columns, a precipitation solution, which consisted of 500 mM CaCl_2_ and 500 mM urea, was introduced under continuous flow conditions. It was shown that hydraulic conductivity was decreased by formation of microbially induced CaCO_3_ precipitation from between 8.37 * 10^−1^ and 6.73 * 10^−2^ cm/s to between 3.69 * 10^−1^ and 1.01 * 10^−2^ cm/s. The lowest hydraulic conductivity was achieved in porous medium consisting of the smallest glass beads (0.25 mm in diameter) using the highest density of cell suspension (OD_600_ 2.25). The number of the deposited cells differed depending on the glass bead size of the columns. According to the experiments, 7 * 10^−9^ g CaCO_3_ was produced by a single resting cell. The urease activity, which led CaCO_3_ precipitation, depended on presence of high number of cells deposited in the column because the nutrients were not included in the precipitation solution and consequently, the amount of CaCO_3_ precipitated was proportional with the cell number in the column. A mathematical model was also developed to investigate the experimental results, and statistical analysis was also performed.

## Key points

- *Sporosarcina pasteurii*, which is an ecologically friendly bacterium for environmental biotechnology, produces urease to form CaCO_3_ precipitation
- CaCO_3_ precipitation decreases the hydraulic conductivity of porous media
- The urease activity depends on the presence of high number of *Sporosarcina pasteurii*

## Introduction

Microbially induced calcium carbonate (MICP) has been studied by many researchers to develop biotechnology methods such as improving construction materials (Philips et al. 2018; Ramachandran et al. 2001), decreasing hydraulic conductivity of porous media (Eryürük et al. 2015a, b), changing soil properties (DeJong et al. 2010; Chou et al. 2011; Arpajirakul et al. 2021; Gao et al. 2022) increasing oil recovery (Wu et al. 2017), sealing cracks (Chen et al. 2021). The field studies were also conducted because of widespread presence of ureolytic bacteria in the environment (Eryürük et al. 2016; Cheng et al., 2017; Nayanthara et al. 2019). Although there are a number of species which produce CaCO_3_ minerals, *Sporosarcina pasteurii* was used by many researchers widely and there are numerous reports on bacterial CaCO_3_ precipitation by this bacterium (Nemati et al. 2003; Ivanov and Chu 2008; Tobler et al. 2011; Al-Thawadi and Cord-Ruwisch 2012; Yasuhara et al. 2012; Al Imran et al. 2018; Seifian and Berenjian 2019; Ghosh et al. 2019; Jain and Arnepalli 2019) because *Sporosarcina pasteurii* is a Biosafety Level (BSL) 1 bacterium with high urease activity (Eryürük et al. 2015a). The process is based on metabolic activity of the bacteria. The urea is hydrolyzed to ammonia and carbon dioxide by *Sporosarcina pasteurii* due to urease and the formation of ammonia leads to an increase in pH of the environment to induce calcite precipitation when calcium ions are present (Eryürük et al. 2015a, b; Wu et al. 2021). To provide Ca^++^, the hydrolysis of urea in a solution with calcium chloride was used in the researches mostly (Harkes et al. 2010; Okwadha and Li 2010; Hsu et al. 2018; Saricicek et al. 2019). The reaction steps and overall reaction can be identified as (Song et al. 2022);

1 $${\text{N}}{\text{H}}_{2}{\text{CON}}{\text{H}}_{2}+{H}_{2}O\stackrel{urease}{\to }{\text{2N}}{\text{H}}_{3}+{\text{C}}{\text{O}}_{2}$$

2 $${\text{2N}}{\text{H}}_{3}+{2}{\text{H}}_{2}O\to {\text{2N}}{{\text{H}}_{4}}^{+}+{\text{2O}}{\text{H}}^{-}$$

3 $${\text{C}}{\text{O}}_{2}+{\text{O}}{\text{H}}^{-}\to {\text{HC}}{{\text{O}}_{3}}^{-}$$

4 $${\text{CaC}}{\text{l}}_{2}\to {\text{C}}{\text{a}}^{2+}+{\text{2C}}{\text{l}}^{-}$$

5 $${\text{C}}{\text{a}}^{2+}+{\text{HC}}{{\text{O}}_{3}}^{-}+{\text{O}}{\text{H}}^{-}\to {\text{CaC}}{\text{O}}_{3}\downarrow +{H}_{2}O$$

6 $$\text{Overall Reaction}\text{ N}{\text{H}}_{2}{\text{CON}}{\text{H}}_{2}+{\text{CaC}}{\text{l}}_{2}+{2}{\text{H}}_{2}O\to {\text{2N}}{{\text{H}}_{4}}^{+}+{\text{2C}}{\text{l}}^{-}+{\text{CaC}}{\text{O}}_{3}\downarrow $$

The hydraulic conductivity of porous media is expected to be reduced by MICP whose steps are shown above. On the other hand, the physiochemical and biological factors such as pH, temperature, existence of nutrients, concentration of nutrients, concentration of precipitation reagents, and oxygen availability all affect the efficiency of the MICP process (Hammes and Verstraete 2002; Li et al. 2018). One of these factors is the density of cells which is related to urease enzyme (Al Imran et al. 2018) Therefore, it is crucial to understand the effect of cell density on hydraulic conductivity using MICP. In the present work, the columns were packed with glass beads of different diameters and the reduction of hydraulic conductivity was studied introducing different density of cell suspension of resting *Sporosarcina pasteurii* to form CaCO_3_ precipitation and to assess the influence of cell density on decrease in hydraulic conductivity of porous media using MICP.

## Materials and methods

### Inoculum preparation, growth conditions and measurement of OD

The strain used in this study was *Sporosarcina pasteurii* [American Type Culture Collection (ATCC) 11859] to induce calcium carbonate precipitation during experiments. The medium (Tris-YE) for cultures was prepared containing Tris buffer (130 mM,pH 9.0), ammonium sulphate (10 g/L) and yeast extract (20 g/L). For stock culture, solid medium was prepared adding 2% agar per liter of liquid medium. The components of media were autoclaved separately at 121 ℃ for 15 min and mixed before usage (Eryürük et al. 2015a).

To observe the relation between cell density and the hydraulic conductivity, resting cells of *Sporosarcina pasteurii* were provided by inoculation of cells in Tris-YE medium overnight at 30 ℃ with 120 rpm shaking. Then the cells were harvested using centrifugation at 10,000*g for 10 min and rinsed two times using distilled water (Eryürük et al. 2015a, b). Ultimately, the cells were suspended in 100 mL distilled water obtaining the optical densities (OD) of 0.15, 0.75, and 2.25 (abbreviated as OD_600_ 0.15, 0.75, and 2.25) at 600 nm. The OD values were measured using a spectrophotometer (Hitachi U-1900 Spectrophotometer, Tokyo, Japan). The aliquots, which were diluted, were taken from cell suspension to count the number of bacterial cells under the microscope (Olympus BX50WI, Tokyo, Japan). The optical densities of 2.25, 0.75, and 0.15 at 600 nm corresponded to 2.15 * 10^9^ cells/mL, 8.10 * 10^8^ cells/mL, 5.89 * 10^8^ cells/mL, respectively.

### Experimental setup and conditions

**Fig. 1:**
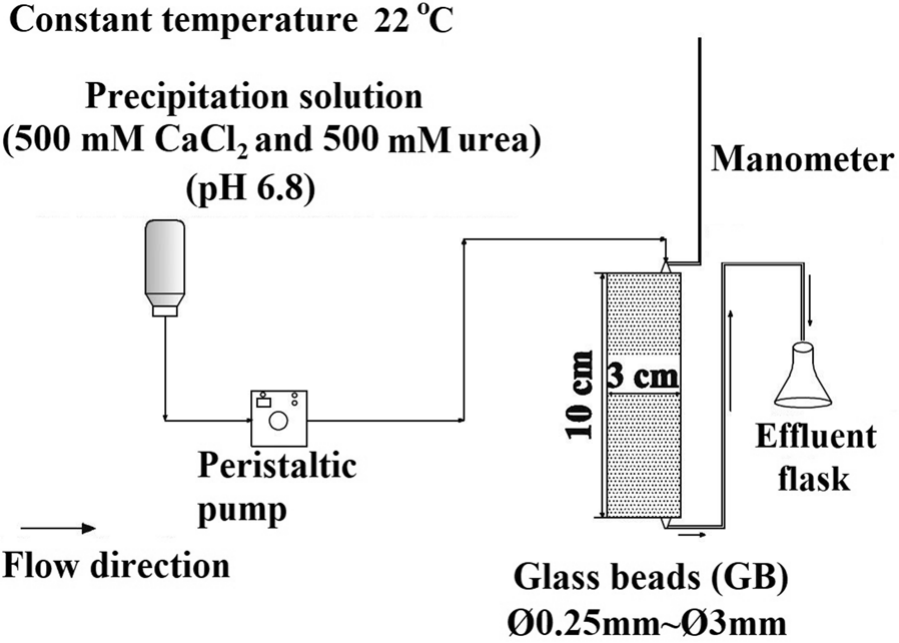
Experimental setup revised from Eryürük et al. 2015a

50 mL-volume of plastic syringes, whose inner diameter was 3 cm inner diameter and height was 10 cm (Fig. 1), were used as columns in the experiments. 90 g of glass beads in average diameter of 0.25 mm, 0.50 mm, 1.0 mm, 2.0 mm, and 3.0 mm were used as the porous media and saturated conditions were provided for each experiment. Before conducting each experiment, an acidic solution (0.1 N HCl) was used to wash the glass beads and then the glass beads were rinsed with distilled water until a neutral pH was achieved.

All experiments were carried out at 22 ℃ as constant temperature and a peristaltic pump controlled the down flow influent rate. First, *Sprosarcina pasteurii* was added into the column as 100 mL of cell suspension (four pore volumes of the packed glass beads) with 2 mL/min of flow rate. Homogeneous cell suspension during introduction of cell suspension was provided using magnetic stirrer. The effluent from the columns was collected to count the number of cells, that flowed out the column, under the microscope (Olympus BX50WI, Tokyo, Japan) and the number of cells deposited in the columns was estimated by subtracting the number of cells eluted from the number of cells introduced to the columns. Then, a 0.22 μm membrane filter was employed to sterilize the precipitation solution which consisted of 500 mM CaCl_2_ and 500 mM urea and had pH 6.8. After that, flow rate of 3 mL/min was used to introduce the precipitation solution (700 pore volumes of the packed glass beads) into the column. The hydraulic conductivity of the column was measured using a manometer. (Eryürük et al. 2015a, b). The experiments were continued by introducing the precipitation solution until no change was measured in the hydraulic conductivity of the columns. All experiments were performed in triplicate.

### Measurement of CaCO_3_ precipitated in the experiments

The porous media material, which was glass beads, was taken into a beaker at the end of the experiments. The glass beads were rinsed two times using distilled water to remove excess Ca^++^. Then, 0.1 N HNO_3_ was added into the beaker to dissolve CaCO_3_ formed by *Sporosarcina pasteurii*. After that, inductively coupled plasma atomic emission spectroscopy (PerkinElmer Optima 3300DV, PerkinElmer, Waltham, Massachusetts, USA) was employed to determine Ca^++^ concentration of the samples which were obtained from the beaker. Finally, detected Ca^++^ concentration was used to calculate the amount of CaCO_3_ precipitated (Eryürük et al. 2015a).

### Measurement of pore size distribution before and after experiments and calculation of occupied volume of CaCO_3_

Matric potential was measured with a wide range pF meter (DIK-3404, Daiki Co., Ltd., Saitama, Japan) to estimate the pore size distribution of the glass beads. The columns were first weighed, and then their bottoms were covered with Advantec No. 6 filter paper (Advantec MFS, Inc., CA, USA). The columns, as well as the ceramic filters of the pF meter, were then saturated with distilled water. The columns were weighed again after 24 h. The ceramic filters and columns were then placed in the pF meter. The pF values 1.3, 1.6, 2.0, 2.5, 3.0, 3.5, and 3.8 were applied by pressure to determine pore sizes of 75–150 μm, 31–75 μm, 10–30 μm, 4–9 μm, 1–3 μm, 0.45–1.00 μm, and < 0.45 μm, respectively. For pF values 1.3–3.0, air was the pressure supplier; for pF values 3.5 and 3.8, nitrogen gas was the pressure supplier. The columns were subjected to pressure for seven days in order to achieve equilibrium. The columns were then reweighed. The pore size distribution of glass beads was then calculated using weight differences between samples after different pF values were applied. The proportion of the individual size ranges of the pores was used to calculate the average pore size in the columns.

The occupied volume of CaCO_3_ was also calculated using specific gravity of CaCO_3_, which is 2.7 g/cm^3^, and the amount of CaCO_3_ precipitation after treatment.

### Analysis of hydraulic conductivity using modified Kozeny-Carman equation

Modified Kozeny-Carman equation (Eryürük et al. 2015a) was used to interpret quantitative relation between the microbial CaCO_3_ precipitation and the reduction of the hydraulic conductivity.

7 $${K}_{Ca+B}\,=\,f\rho {g\left(n-{r}_{Ca}B/V\right)}^{3}/\left[\mu {\left\{6/(6{r}_{Ca}B/\pi +{D}^{3}{)}^{1/3}\right\}}^{2}(1-n+{r}_{Ca}B/V{)}^{2}\right]$$

where K_Ca+B_ denotes hydraulic conductivity with bacterial clogging, f denotes the shape factor, ρ denotes the density of liquid, g denotes gravity, n denotes porosity, r_Ca_ denotes the specific CaCO_3_ precipitation rate, B denotes the number of cells deposited, V denotes the volume of column, µ denotes the dynamic viscosity of the liquid, and D denotes the average diameter of glass beads.

## Mathematical model for variables of experiments

15 experimental trials were employed in a random order in accordance with the optimal (custom) design, which allows for a flexible design structure to accommodate custom models, categorical factors, and irregular (constrained) regions.

The results were obtained using the software Design-Expert 11.0.5.0 (Stat-Ease Inc., Minneapolis, MN, USA, 2021) which can be used to investigate the relationship between multiple input variables and key output variables (Eryürük et al. 2021). In this study, the independent variables were determined as OD_600_ (A), glass beads size (B), CaCO_3_ (C), and deposited cell number (D). The dependent factor, hydraulic conductivity (K), was the response. OD_600_ (A) is a discrete variable with three levels; 0.15, 0.75 and 2.25. Glass beads size (B) is a discrete variable with five levels; 0.25, 0.5, 1, 2, 3. CaCO_3_ (C) is a continuous variable having values between 2.981 and 15.313. Deposited cell number (D) is a continuous variable having values between 2.38E+07 and 3.51E+09.

## Results

**Fig. 2:**
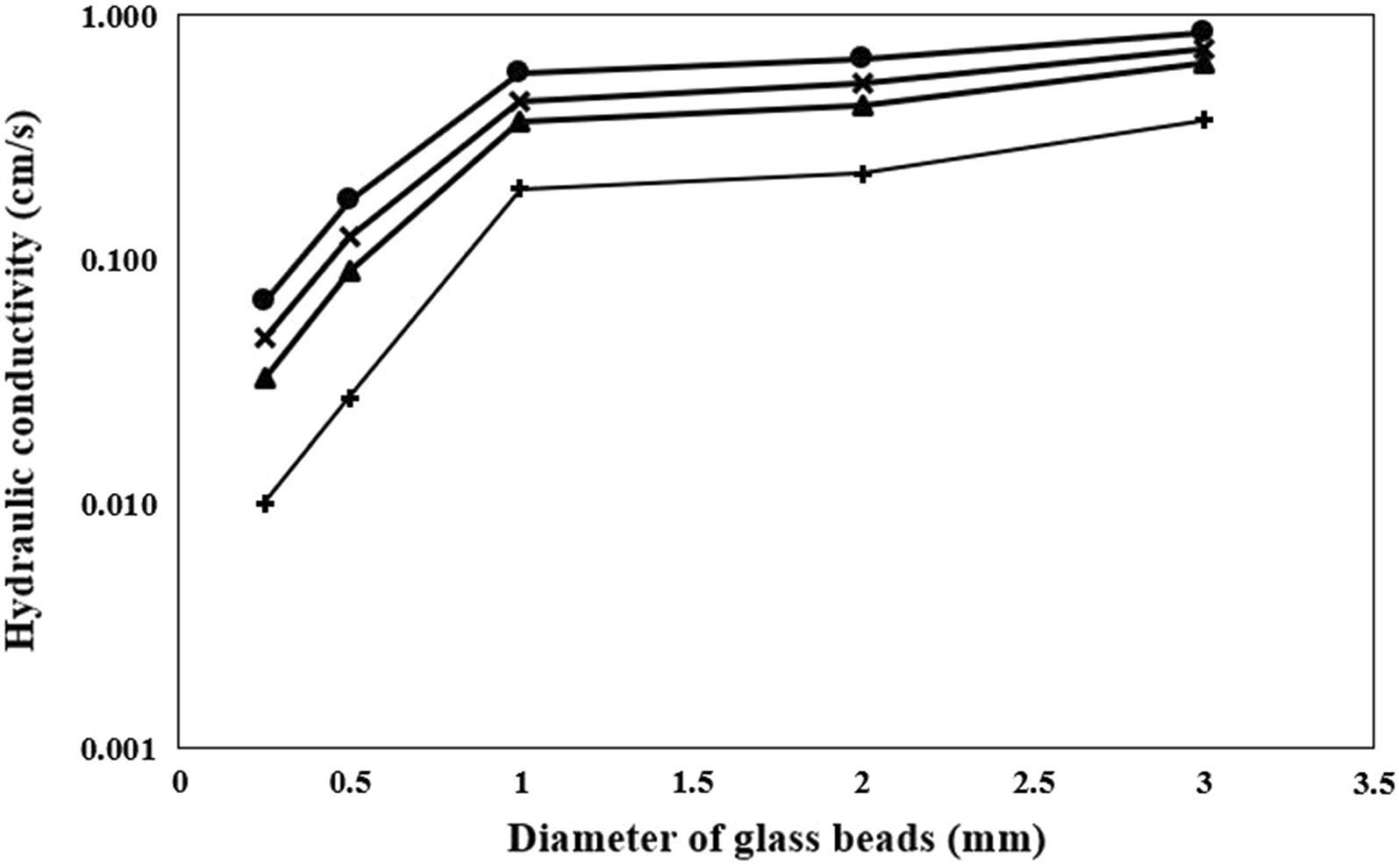
Hydraulic conductivities of glass beads before treatment (closed circle) and after treatment using 500 mM of CaCl_2_ and 500 mM urea, and cell suspension as OD_600_ 0.15 (cross sign), OD_600_ 0.75 (closed triangle), OD_600_ 2.25 (plus sign)

**Fig. 3:**
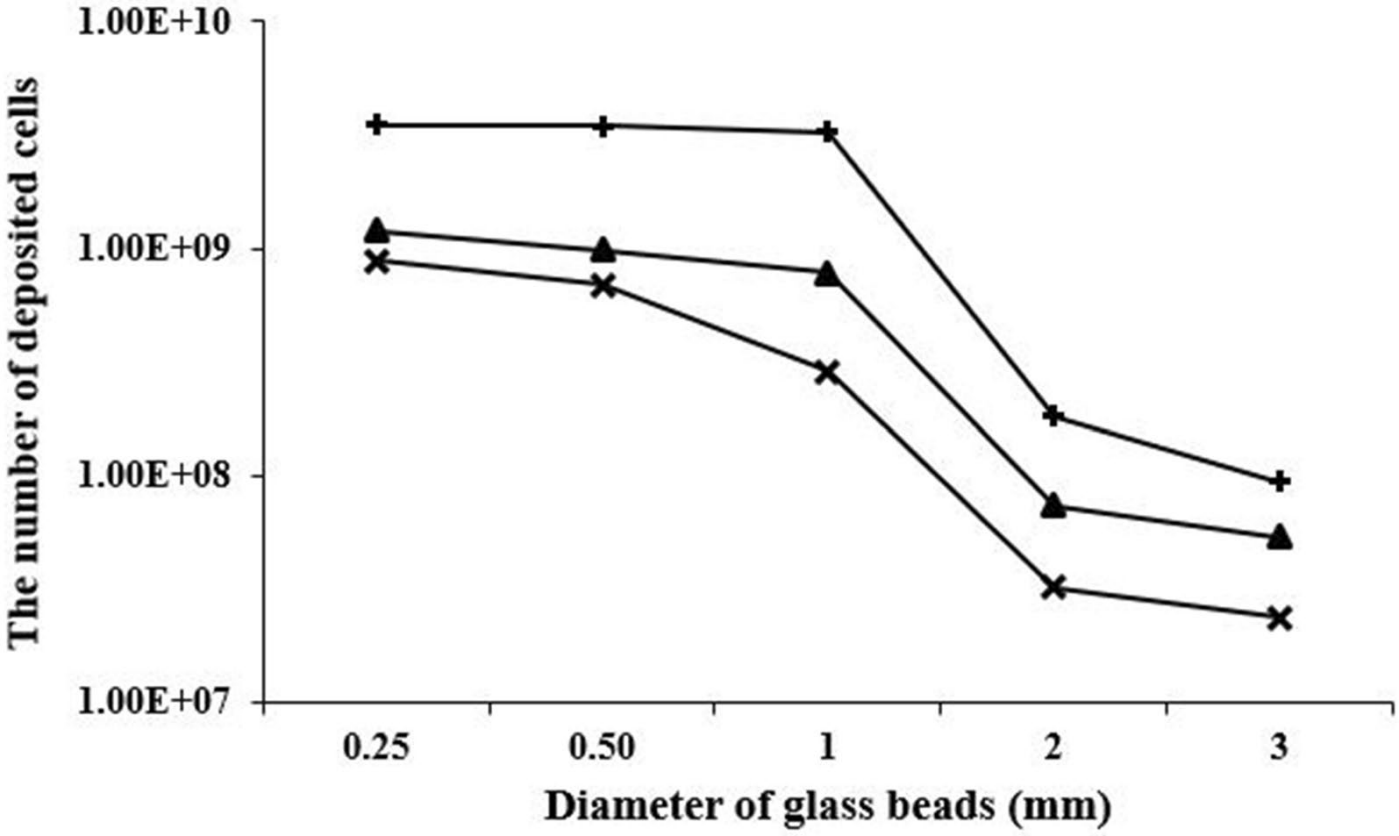
The number of cells deposited into the columns packed with different diameter of glass beads for OD_600_ 0.15 (cross sign), OD_600_ 0.75 (closed triangle), OD_600_ 2.25 (plus sign)

**Fig. 4:**
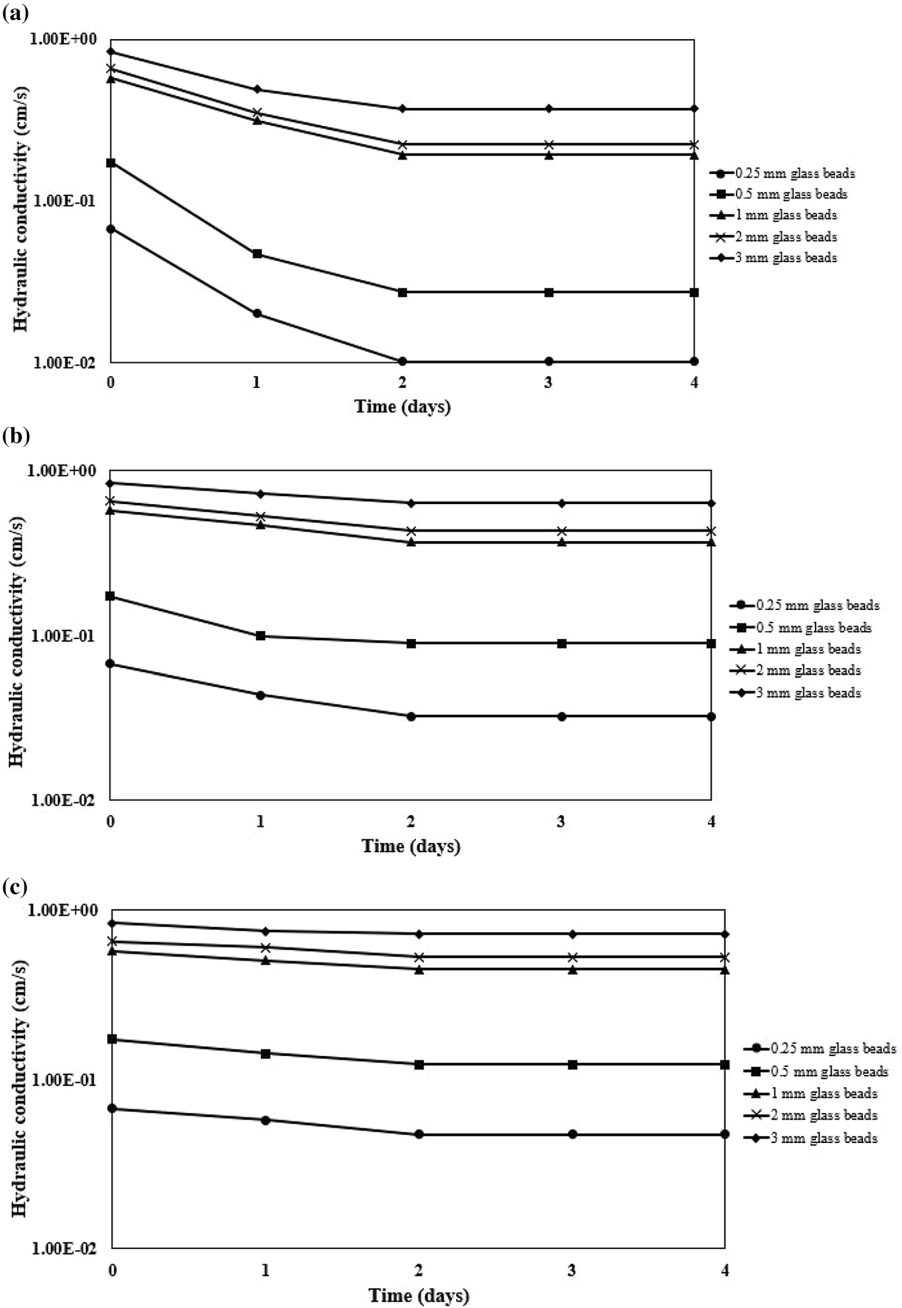
Change in hydraulic conductivity in time for the experiments conducted with **a** OD_600_ 2.25, **b** OD_600_ 0.75, and **c** OD_600_ 0.15

The measurement of the hydraulic conductivities of glass beads column were performed as shown in Fig. 2. The initial hydraulic conductivity was measured as 6.73 * 10^−2^ cm/s, 1.72 * 10^−1^ cm/s, 5.74 * 10^−1^ cm/s, 6.55 * 10^−1^ cm/s, and 8.37 * 10^−1^ cm/s for columns containing 0.25 mm, 0.50, 1.0 mm, 2.0 mm, and 3.0 mm glass beads, respectively. The lowest hydraulic conductivity was observed for all columns when the experiments conducted with OD_600_ 2.25 which has the highest concentration of cells (Fig. 3). It can also be said that continuously introduction of precipitation solution led in reduction of hydraulic conductivity of the columns (Fig. 2). However, the hydraulic conductivity became stable, which was attributed to using resting cells of *Sporosarcina pasteurii* without any nutrient source, subsequently 2 days of injection of the precipitation solution that equaled to 350 pore volumes (Fig. 4a–c).

Figure 3 also indicates that the highest number of cells were deposited using OD_600_ 2.25. The number of cells deposited also increased when the small size of glass beads in diameter was packed in columns. The number of the deposited cells changed depending on the glass beads diameter, probably because of difference of the pore size, and in relation to the cell density of the introduced bacterial suspension.

**Fig. 5:**
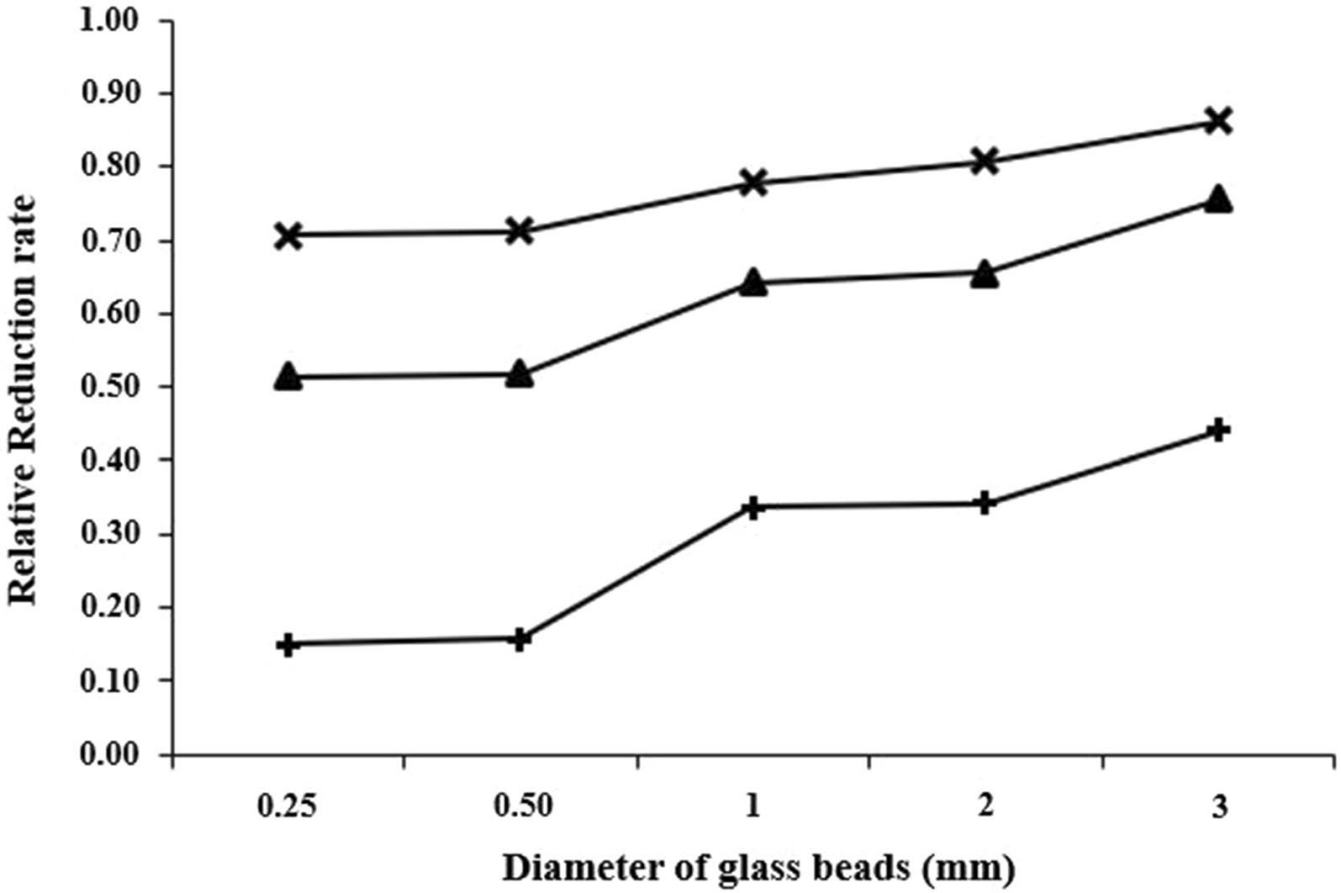
Relationship between cell densities of OD_600_ 0.15 (cross sign), OD_600_ 0.75 (closed triangle), OD_600_ 2.25 (plus sign) introduced into the column and the proportion of the hydraulic conductivity of the treated column to the initial hydraulic conductivity

As shown in Fig. 5, the highest conductivity reduction was observed when the smallest diameter of glass beads (0.25 mm glass beads) were packed into the columns and the density of the cell suspension was OD_600_ 2.25. The lowest reduction was measured in the columns packed with 3.0 mm glass beads using the lowest density of cells (OD_600_ 0.15). The reduction was enhanced by increase in the cell density of the cell suspension. The relative decrease rate of hydraulic conductivity was determined by the number of cells deposited which was related to urease activity.

**Fig. 6:**
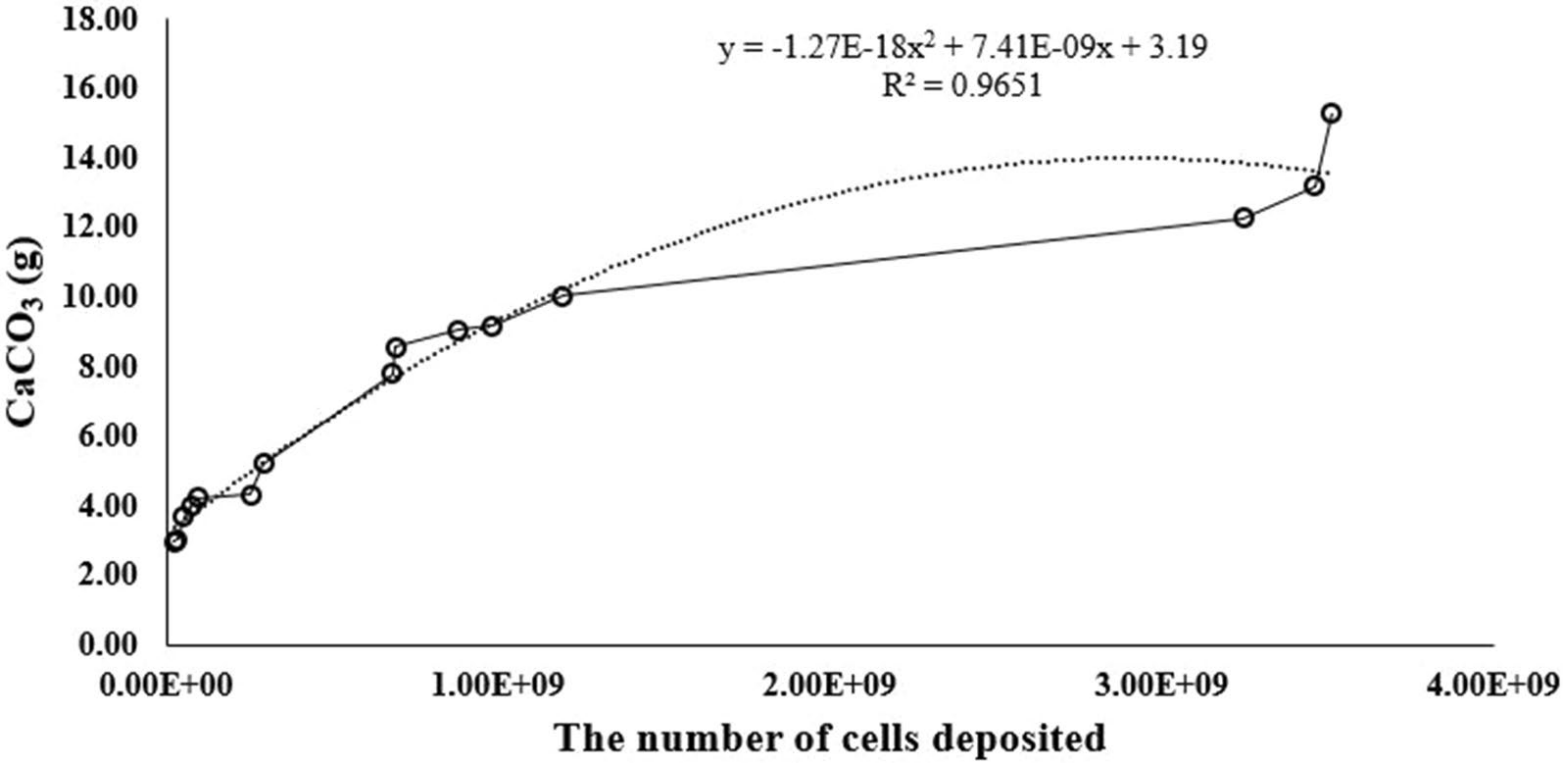
The number of cells deposited and the amount of CaCO_3_ precipitated

When the numbers of cells deposited in the columns were 10^8^ and 10^7^ cells, the amount of CaCO_3_ precipitated did not exceed 5 g after the experiments. (Fig. 6). When numbers of cells deposited in the columns were greater than 10^9^ cells, the amount of CaCO_3_ precipitated was increased to more than 10 g after the experiments (Fig. 6). The amount of CaCO_3_ precipitated was proportional to cell numbers in the column (Fig. 6) with the surprising result that a single *Sporosarcina pasteurii* cell (dry mass ~ 6 * 10^–13^ g) could produce roughly 10,000 times that mass of calcium carbonate (7 * 10^–9^ g) per cell, and that the linear dimension of the average calcite crystal surrounding a single cell might be an order of magnitude larger than the cell itself.

For the experiments conducted with OD_600_ 2.25, which produced the largest amount of CaCO_3_ precipitation and obtained the lowest hydraulic conductivity, the average pore size in the columns were evaluated. CaCO_3_ precipitation resulted in change in the average pore size in the treated columns from 66.1 μm to 51.7 μm, 75.8 μm to 61.8 μm, 77.6 μm to 63.1 μm, 91.7 μm to 75.4 μm, and 96.8 μm to 79.3 μm for the columns packed with 0.25 mm, 0.50 mm, 1 mm, 2 mm, and 3 mm glass beads in diameter, respectively. The pore volume, which was 25 cm^3^ for all the columns, were reduced 19.3 cm^3^, 20.1 cm^3^, 20.5 cm^3^, 23.1 cm^3^, 23.4 cm^3^ for the columns included 0.25 mm, 0.50 mm, 1 mm, 2 mm, and 3 mm glass beads in diameter, respectively.

**Fig. 7:**
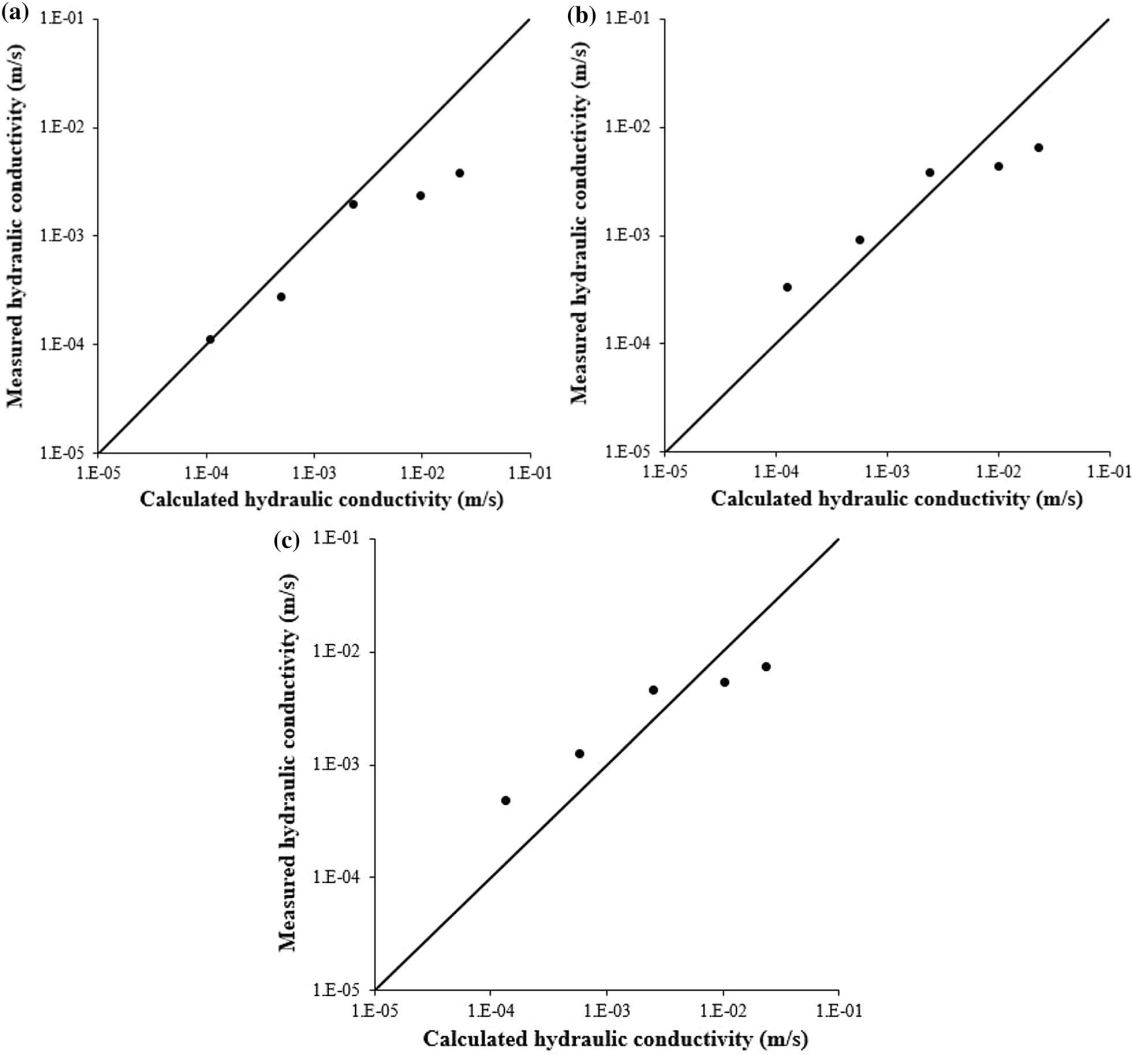
Estimated hydraulic conductivity using modified Kozeny-Carman equation and measured hydraulic conductivity for **a** OD_600_ 2.25, **b** OD_600_ 0.75, and **c** OD_600_ 0.15

As shown in Fig. 7a–c, modified Kozeny-Carman equation gave a good agreement between the calculated values and the measured ones in hydraulic conductivity. Analysis using modified Kozeny–Carman equation indicated that CaCO_3_ precipitate aggregated with the glass beads and increased the beads’ effective size to decrease the hydraulic conductivity.

The model is likely significant given the model F-value of 88.50 and model p-value of < 0.0001. If the model p-value is less than 0.05, the model terms are statistically significant. OD_600_ (A), glass beads size (B), CaCO_3_ (C), and deposited cell number (D) are significant model terms having value p-value of 0.0001, 0.0003, 0.0043 and 0.0007 in this study respectively. p-value greater than 0.10 indicates that the model terms are not significant.

The design's performance was evaluated by examining the minimization of hydraulic conductivity. The current evaluation of the mathematical model's significance at a 95% confidence interval used the analysis of variance (ANOVA) test. The adjusted R^2^ of 0.9615 is quite consistent with the predicted R^2^ of 0.9042 because the difference is less than 0.2, and the R^2^ value is 0.9725.

The mathematical model is as follows:

$$ \begin{aligned} {\text{K }}\left( {{\text{cm}}/{\text{s}}} \right) \, & = \, 0.{44}0{827 } - \, 0.{131344 }*{\text{OD}}_{{{6}00}} \left( {\text{A}} \right) + \, 0.{15723}0 \, *{\text{Glass beads size }}\left( {\text{B}} \right) - \, 0.0{57997} \\ & \quad\, *{\text{CaCO}}_{{3}} \left( {\text{C}} \right) + { 1}.{\text{89632E}} - {1}0 \, *{\text{Deposited cell number }}\left( {\text{D}} \right) \\ \end{aligned} $$

**Fig. 8:**
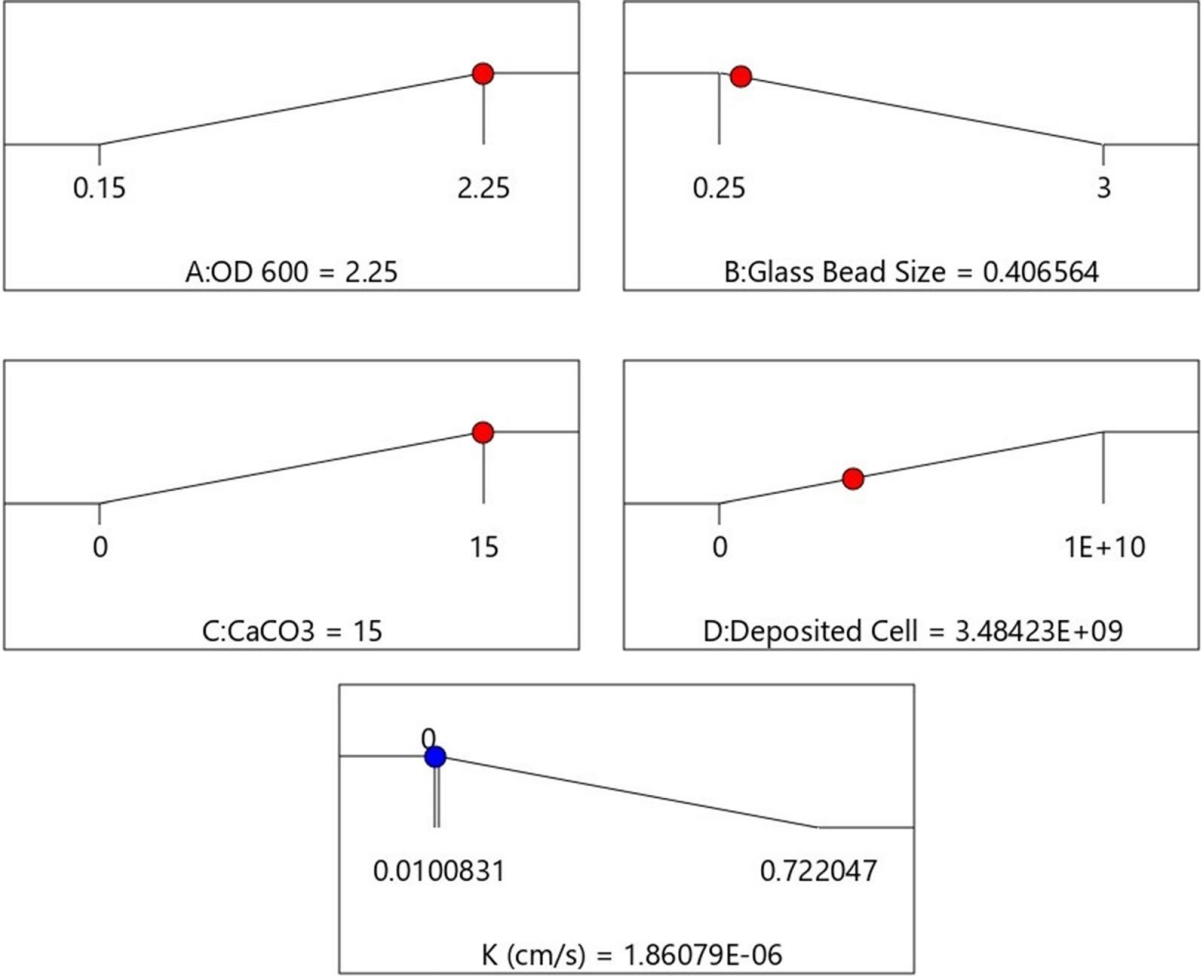
Optimal conditions

In this study, the linear mathematical model is significant. By optimizing the ideal factor settings, the goal of reducing hydraulic conductivity (Y) value can be achieved. Ideal factor values and the optimal response value is given at Fig. 8.

**Fig. 9:**
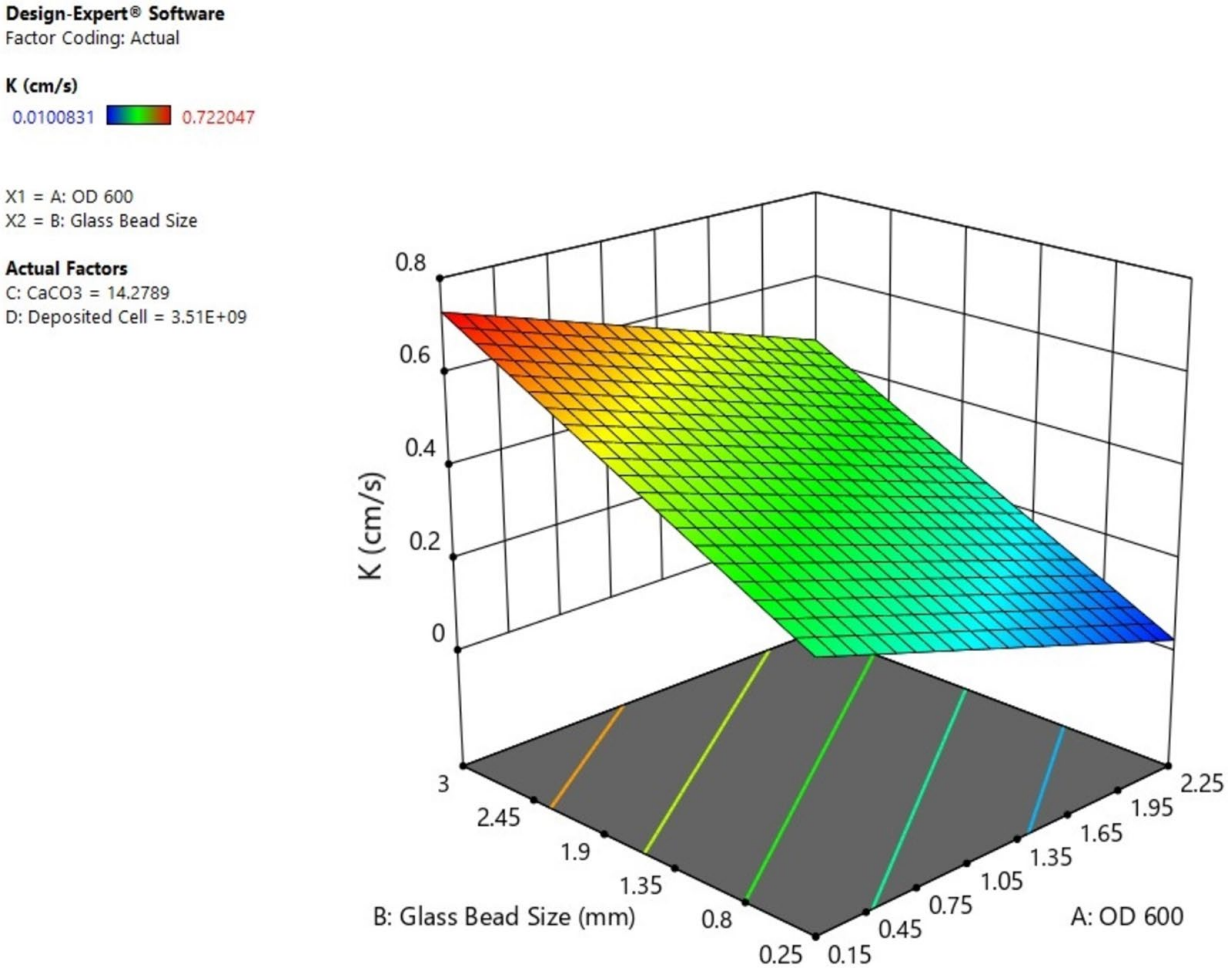
Surface plot of OD_600_ (A), glass beads size (B)

There is a linear relationship between hydraulic conductivity (K), OD_600_ (A), glass beads size (B), CaCO_3_ (C), and deposited cell number (D). A three-dimensional surface plot of hydraulic conductivity (K) versus OD_600_ (A) and glass beads size (B) while changing value of CaCO_3_ (C), and deposited cell number (D) can be seen in Fig. 9. Moreover, there is no nonlinear relationship between hydraulic conductivity (K) and other factors.

## Discussion

Generally, MICP was investigated by addition of bacteria in diluted spent growth medium with no guarantee that the dilute organics still present cannot support further multiplication (Seifan and Berenjian 2018; Chuo et al. 2020; Feng et al. 2021) However, this study offers a virtually unique opportunity to measure the contribution of single cells to CaCO_3_ deposition by careful washing and introduction of only resting cells. Thus, the aim of the present study could be identified as investigating the effect of cell density on decrease in hydraulic conductivity using MICP by resting cells. OD_600_ 0.15, OD_600_ 0.75, and OD_600_ 2.25 were used to provide different cell densities. The columns were packed with different size of glass beads in diameter to have porous media.

The lowest hydraulic conductivity was achieved by OD_600_ 2.25 in this study as 6.73 * 10^–2^ cm/s which was measured in the column with packed with 0.25 mm diameter of glass beads. The highest amount of CaCO_3_ was also measured by OD_600_ 2.25 in the experiments with 0.25 mm diameter of glass beads.

The hydraulic conductivity did not change after 2 days injection of precipitation solution for all experiments. Eryürük et al. (2015a) suggested that glass beads, *Sporosarcina pasteurii* cells and CaCO_3_ form a single particle. Depending on this suggestion, the halo of alkalinity around each *Sporosarcina pasteurii* cell would be diluted to the point that additional calcite was no longer deposited on the periphery of each deposit.

Omoregie et al. (2019) and Maleki-Kakelar et al. (2022) claimed that the addition of nutrient source results in enhancement in urease activity of *Sporosarcina pasteurii*. Therefore, the urease activity generated by cells in the columns would be exhausted probably because there was no nutrient source in the precipitation solution. Graddy et al. (2021) investigated feeding *Sporosarcina pasteurii* in natural sands. The results indicated that native species of *Sporosarcina* quickly outcompeted feeding cells (Graddy et al. 2021) most probably they are spore formers, and spores are difficult to kill even by autoclaving.

When the mechanism of decrease in hydraulic conductivity was investigated, the results indicated that the glass beads, cells, and CaCO_3_ precipitate most probably aggregated into spherical particles instead of remaining as separate, individual particles of glass beads, bacteria, and CaCO_3_ (Eryürük et al. 2015a). In this study, analysis using modified Kozeny-Carman equation (Eryürük et al. 2015a) suggested that increases in the effective size of packing glass beads with microbially induced CaCO_3_ precipitation resulted in a decrease in the hydraulic conductivity of the column due to decreases in the specific surface area and porosity of the porous media.

In conclusion, this study achieved to decrease the hydraulic conductivity of porous media using MICP by resting cells of *Sporosarcina pasteurii*. The results highlighted the total activity of urease introduced into the column, and thus the amount of CaCO_3_ precipitation, was determined by the number of cells deposited in the column. Groundwater contamination, which affects drinking water resources due to leakage from landfills, agricultural activities, and other sources, is a crucial problem all over the world (Sasakova et al. 2018; Ma et al. 2021). This study simulated a nutrient-deficient environment using resting cells without nutrients. Considering field application to achieve lower hydraulic conductivity for providing an impermeable layer in the soil, bacterial growth could be investigated using addition of nutrient source in precipitation solution for future study. Furthermore, the model developed for this study could also be improved and optimization and validation experiment could be investigated for future study.

## Data Availability

The authors promise the availability of data and materials.
